# Subgroup analyses and effect modification with Bayesian kernel machine regression

**DOI:** 10.1093/aje/kwaf281

**Published:** 2025-12-19

**Authors:** Danielle Demateis, Kaleigh P Keller, Brent A Coull, Ander Wilson

**Affiliations:** Department of Statistics, Colorado State University, Fort Collins, CO, United States; Department of Statistics, Colorado State University, Fort Collins, CO, United States; Department of Biostatistics, Harvard T.H. Chan School of Public Health, Boston, MA, United States; Department of Statistics, Colorado State University, Fort Collins, CO, United States

**Keywords:** Bayesian kernel machine regression, environmental mixtures, effect modification, effect heterogeneity, subgroup analyses

## Abstract

There is substantial interest in estimating the health effects of exposure to environmental mixtures. Bayesian kernel machine regression (BKMR) has emerged as a popular tool for mixture analyses. The health effects of environmental exposures, including mixture exposures, often differ among subpopulations. However, there is little guidance on how to assess such heterogeneity for mixture effects. We provide tools and guidance to conduct BKMR analyses with effect modification, including estimating group-specific effects and between-group differences in effects. We propose a new group-separable BKMR variant for mixture analyses with effect modification by a categorical variable. We compare this new method to a stratified analysis and to a model that includes the categorical modifier directly in the BKMR kernel function in both a simulation study and the analysis of a metals mixture on children's neurodevelopment with child sex as a binary modifier in a rural Bangladesh cohort. Both stratified BKMR and the new group-separable BKMR have the flexibility to capture interactions and estimate between-group differences. The group-separable BKMR has lower variance compared to stratified BKMR, particularly when there are small subgroup sizes. We provide code and data to implement the methods and reproduce simulations and analyses.

## Introduction

There is widespread interest in understanding how exposure to environmental mixtures impacts human health.^1-3^ Challenges inherent to the epidemiological analysis of environmental mixture exposures include high correlation among components in a mixture, high-dimensionality of the mixture, and complex exposure–response relationships, including interactions between pollutants and non-linear associations with the response.^4^ To address these and other challenges, numerous methods have been proposed for the analysis of environmental mixtures data.^4-11^ Several reviews have characterized methods that can address common research goals in mixture analyses, including identifying toxic pollutants, estimating the effects of individual pollutants and/or the whole mixture on a health response, and identifying interactions between pollutants.^5^^,^^7^^,^^9^^,^^12^ However, a notable gap in the literature is guidance on assessing effect modification in mixtures analyses.

There are many examples of the scientific importance of effect modification of an environmental mixture in the literature. For example, it is commonly hypothesized that sex-specific differences in fetal development may lead to sexually dimorphic effects of exposure.^13-16^ Bhatt et al.^13^ provides multiple biological mechanisms by which the effect of exposure may differ by boys and girls, suggesting that identification of such sex-specific effects could provide insights into these mechanisms. Other examples include effect modification by demographic factors,^17^^,^^18^ which can identify subpopulations that are particularly susceptible and inform population-specific interventions. Another important goal is to identify factors that may protect against the effects of mixture exposure, again informing potential intervention strategies. Recent studies in this category involving mixtures include those focusing on effect modification by nutrients^19^^,^^20^ and built environment.^21^

When estimating the association between a single environmental pollutant and a health outcome, it is common to include an interaction term in a parametric regression model to allow for different effects among subgroups. Methods for effect modification have been implemented for linear index models.^16^^,^^22-25^ However, modeling effect modification is more complicated with sophisticated statistical methods for mixture analyses, including high-dimensional exposure–response surface methods. This is especially true for modification by a categorical factor, such as child sex or smoking status,^19^^,^^26-31^ as most statistical approaches for mixture analyses are designed for multiple continuous predictors and may not be appropriate for binary or categorical factors. Therefore, it is common for researchers to fit models stratified by a categorical variable that may alter the association between the exposures and response. However, stratified analyses are limited by decreased power due to small within-group sample sizes and the challenge to quantify differences in exposure effects between groups and the uncertainty associated with these difference estimates. Thus, there is a need for analysis methods that specifically allow for modification by categorical factors in mixtures analyses. Bayesian kernel machine regression (BKMR) is a widely used method for mixture analyses in epidemiological studies.^32^^,^^33^ BKMR is a semi-parametric regression method that can estimate a flexible, multi-dimensional exposure–response surface. To allow for complex associations while also reducing overfitting, BKMR uses a kernel function in a hierarchical Bayesian framework to estimate the exposure–response surface. While BKMR can address many common research questions for environmental mixtures, there is currently no guidance in the literature on how to allow for modification by a categorical factor. Several studies have applied BKMR separately to different subgroups in stratified analyses.^26^^,^^27^^,^^29-31^ Alternatively, it is feasible to include a categorical factor in the kernel function to assess modification.^32^ However, this approach has not been rigorously evaluated.

We consider BKMR analyses of environmental mixtures with modification by a categorical variable. We consider three approaches, including two that rely on the standard BKMR model and software and a new variant introduced in this paper. We evaluate the operating characteristics of each method through a simulation study to give practical advice on how to conduct a mixture analysis using BKMR with effect modification by a categorical factor. We also illustrate the approaches through an analysis of a metal mixture and infant neurodevelopment from a Bangladesh cohort. We make code available to implement all methods in other studies in a new R package and provide the code and data necessary to reproduce our simulation and data analysis results.

## Methods

### Statistical methods

For individual $i=\mathrm{1,}\dots, n$, let ${y}_i$ be a continuous response, ${\mathbf{z}}_i=\left[{z}_{i1},\dots, {z}_{iM}\right]{}^{\prime }$ be a vector of $M$ different continuous exposures in a mixture, ${\mathbf{x}}_i$ be a vector of covariates, and ${w}_i$ be a categorical variable with $P$ categories. We denote the levels of the modifier as ${w}_i\in \mathrm{\{1,}\dots, P\}$ and $\mathbf{w}=\left[{w}_1,\dots, {w}_n\right]{}^{\prime }$.

#### Standard BKMR

The standard BKMR model^33^ with no effect modification is


$$ {y}_i=h\left({\mathbf{z}}_i\right)+\mathbf{x}{\hbox{'}}_i\beta +{\varepsilon}_i, $$

where $h\left({\mathbf{z}}_i\right)$ is a nonparametric exposure–response function for individual $i$ that may include nonlinearities and interactions, $\beta$ is a vector of regression coefficients for the covariates, and ${\varepsilon}_1,\dots, {\varepsilon}_n$ are residual terms that are assumed to be independent and normally distributed with mean zero and variance ${\sigma}^2$.

BKMR parameterizes the exposure–response function $h\left(\cdot \right)$ with a Gaussian process. This allows for a high-dimensional set of predictors and induces smoothness and allows for non-linear associations and interactions. For a vector of outcomes $\mathbf{y}=\left[{y}_1,\dots, {y}_n\right]{}^{\prime }$, matrix of exposures $\mathbf{Z}=\left[{\mathbf{z}}_1,\dots, {\mathbf{z}}_n\right]{}^{\prime }$, and matrix of covariates $\mathbf{X}=\left[{\mathbf{x}}_1,\dots, {\mathbf{x}}_n\right]{}^{\prime }$, BKMR can be expressed as the hierarchical model


$$ \mathbf{y}\mid \mathbf{h},\beta, {\sigma}^2\sim N\left(\mathbf{h}+\mathbf{X}\beta, {\sigma}^2\mathbf{I}\right) $$

$$ \mathbf{h}\mid \rho, \tau \sim N\left[\mathbf{0},\tau \mathbf{K}\right(\mathbf{Z},\rho \mathrm{\left)\right],} $$

where $\mathbf{h}=\left[{h}_1,\dots, {h}_n\right]{}^{\prime }$ is the exposure–response vector, $\mathbf{K}\left(\mathbf{Z},\rho \right)$ is a covariance matrix defined by a kernel function of the exposures, $\rho =\left[{\rho}_1,\dots, {\rho}_M\right]{}^{\prime }$ is a vector of parameters that control the smoothness of the exposure–response surface separately in each exposure dimension, and $\tau$ is a variance component that controls the overall smoothness of the exposure–response surface. BKMR is most commonly applied with a Gaussian kernel function. The $(ij)$ element of the Gaussian kernel matrix $\mathbf{K}\left(\mathbf{Z},\rho \right)$ is


$$ K\left({\mathbf{z}}_i,{\mathbf{z}}_j,\rho \right)=\exp \left[-\sum \limits_{m=\mathrm{1}}^M\frac{\Big({z}_{im}-{z}_{jm}{\Big)}^2}{\rho_m}\right]. $$

A standard BMKR model can be fit with Markov chain Monte Carlo (MCMC) methods using the bkmr R package.^34^

#### Stratified BKMR

The stratified approach to effect modification in BKMR fits a separate BKMR model to data containing only individuals having a given level of the modifier $w$, yielding different exposure–response surfaces for each group. Table 1 summarizes the key model assumptions of this method and the others discussed herein. This stratified approach to estimating effect modification assumes no sharing of information among groups, which limits inference. Most importantly, the exposure–response surfaces are estimated independently for each group, so the exposure–response surface is smoothed differently for each group, and there is no penalization or sharing of information that imposes a similar shape or smoothness in the surfaces among the groups. In addition, the effects of covariates ($\beta$) and error variance (${\sigma}^2$) are different for each group. When fitting a stratified analysis, the modifier is not included in the covariates because there is no variation in that variable within groups.

**Table 1:** Assumptions for models with modification considered in this manuscript: Modifier-in-kernel (mod-in-kernel), group-separable, and stratified. The  symbol indicates that the model does have, and the X symbol indicates that the model does not have, the corresponding assumption.

**Assumptions**	**Mod-in-Kernel**	**Group-Separable**	**Stratified**
Pooled analysis			X
Parameters ${\sigma}^2$ and $\beta$ shared among groups			X
Exposure-specific smoothing parameters ($\rho$) shared among groups			X
Overall exposure–response surface smoothing parameter ($\tau$) shared among groups		X	X
Penalty on exposure–response shape among groups		X	X

#### Modifier-in-kernel BKMR

The second approach we consider is to include the modifier in the kernel along with the exposures. This approach can also be estimated with the existing BKMR package.^34^ When there are more than two levels to the categorical modifier, and it is not assumed to be ordinal, the modifier should first be converted to indicator variables for group membership. Let ${\mathbf{d}}_i=\left[{d}_{i1},\dots, {d}_{i(P-\mathrm{1}\mathrm{)}}\right]{}^{\prime }$ contain indicator variables for modifier ${w}_i$ with one group omitted as the reference group. We append ${\mathbf{d}}_i$ to ${\mathbf{z}}_i$ and fit a standard BKMR. The ($ij$) element of the kernel matrix is then


\begin{align*} &{K}^{\mathrm{mk}}\left({\mathbf{z}}_i,{\mathbf{z}}_j,{\mathbf{d}}_i,{\mathbf{d}}_j,{\rho}^{\mathrm{mk}}\right)\\&\qquad=\exp \left[-\left\{\sum \limits_{m=\mathrm{1}}^M\frac{\Big({z}_{im}-{z}_{jm}{\Big)}^2}{\rho_m^{\mathrm{mk}}}+\sum \limits_{p=\mathrm{1}}^{P-1}\frac{\Big({d}_{ip}-{d}_{jp}{\Big)}^2}{\rho_{p+M}^{\mathrm{mk}}}\right\}\right]. \end{align*}

The superscript “mk” is placed on parameters in this model to denote the modifier-in-kernel BKMR model. The indicator variables ${\mathbf{d}}_i$ for each individual would typically be included in the model as covariates.

Of the methods discussed here, this approach makes the strongest assumptions about sharing information among levels of the modifier (Table 1). This approach shares the same smoothing parameters (${\rho}_1^{\mathrm{mk}},\dots, {\rho}_{M+P-1}^{\mathrm{mk}}$ and $\tau$) for all subgroups, resulting in approximately the same level of smoothness for the exposure–response surface in each group. A defining characteristic of this model is that the exposure–response surface has an additional dimension for each indicator variable. This model imposes a penalty that induces similarity in the exposure–response function between each group and the reference group. Additionally, this method shares the same regression coefficients and error variance between groups.

#### Group-separable BKMR

The new approach for effect modification with BKMR that we present here uses a separable kernel function. This allows for a level of information sharing among groups between that of the stratified and modifier-in-kernel approaches. The model can be fit with the bkmrGS package that uses a similar MCMC algorithm as the standard BKMR model. The prior on the exposure–response surface is


$$ {\mathbf{h}}_{\mathbf{w}}^{\mathrm{gs}}\mid{\rho}^{\mathrm{gs}},{\tau}^{\mathrm{gs}}\sim N\left[\mathbf{0},{\mathbf{K}}^{\mathrm{gs}}\right(\mathbf{Z},\mathbf{w},{\rho}^{\mathrm{gs}},{\tau}^{\mathrm{gs}}\mathrm{\left)\right],} $$

where ${\tau}^{\mathrm{gs}}$ is a vector of length $P$ containing a unique smoothing parameter for each modifier group. The kernel function for the ($ij$)-element of the kernel matrix ${\mathbf{K}}^{\mathrm{gs}}\left(\mathbf{Z},\mathbf{w},{\rho}^{\mathrm{gs}},{\tau}^{\mathrm{gs}}\right)$ is


$$ {K}^{\mathrm{gs}}\left({\mathbf{z}}_i,{\mathbf{z}}_j,{w}_i,{w}_j,{\tau}^{\mathrm{gs}},{\rho}^{\mathrm{gs}}\right)\!=\!\left\{\!\!{\displaystyle \begin{array}{cc}{\tau}_{w_i}^{\mathrm{gs}}\times \exp \left[\!-\sum \limits_{m=\mathrm{1}}^M\frac{\Big({z}_{im}-{z}_{jm}{\Big)}^2}{\rho_m^{\mathrm{gs}}}\right],& \!\!\!{w}_i={w}_j\\ 0,&\!\!\! {w}_i\ne{w}_j\end{array}}\right.\!\!, $$

where ${\tau}_{w_i}^{\mathrm{gs}}$ is the smoothing parameter for modifier group ${w}_i$ for individual $i$. The superscript “$\mathrm{gs}$” is placed on parameters in this model to denote the group-separable approach. The kernel matrix has a block-diagonal structure, meaning that the matrix is constructed with a group of non-zero elements for each modifier level and with blocks of zeros for the correlations between observations with different levels of the modifier. This removes the penalty that induces similarity in the exposure–response surface among the different subgroups. This approach also allows for different levels of smoothness in the exposure–response surface for each level of the modifier by letting the smoothing parameter $\tau$ vary among the subgroups. It reduces variance through parameter sharing of $\beta$, ${\sigma}^2$, $\rho$. The indicator variables ${\mathbf{d}}_i$ for each individual would typically be included in the model as covariates ($\mathbf{X}$).

## **Interpretation of the exposure**–**response functions**

There are multiple types of exposure–response effects that are of interest in a BKMR model. We focus on two commonly used summaries of the exposure–response surface for BKMR as previously described^32^: the total effect of the mixture and single-exposure effects. The total effect of the mixture on the response is the difference in expected outcome when all exposures are fixed at one quantile (${q}_2$) compared to when all exposures are fixed at a different quantile (${q}_1$) (eg, ${q}_2=\mathrm{0.75}$, ${q}_1=\mathrm{0.25}$). The single-exposure effect of a given exposure on the response is the difference in expected outcome when one exposure is fixed at the ${q}_1$ quantile compared to when it is fixed at the ${q}_2$ quantile and all other exposures are fixed at quantile $q$ (eg, $q=\mathrm{0.50}$). For modification, these parameters are estimated separately for each level of the modifier, and between-group differences are calculated as the difference between levels. Full details and generalized definitions are reported in Appendix S1.

### Bangladesh reproductive cohort data

#### Data description

We obtained data from The Bangladesh Reproductive Cohort Study (Project Jeebon) for 351 children born between 2008 and 2013 in the Pabna Upazilas (administrative region) of Bangladesh. The data have been previously described and made publicly available in a partially masked dataset.^35^ We consider a mixture of cumulative exposure to three metals during gestation measured using umbilical cord venous blood samples at delivery: lead, manganese, and arsenic. The dependent variable in our analysis is infant neurodevelopment quantified using the Bayley Scales of Infant and Toddler Development, Third Edition (BSID-III)^36^ measured at 20 to 40 weeks of infant age. Specifically, we use the fine motor domain score from BSID-III where higher scores indicate higher cognitive performance in the domain. We consider child sex as a single binary modifier in our analyses. The data also contain the following covariates for which we adjusted in all analyses: parent's demographic status (age, education, smoking history), infant's biometric measurements at birth (sex, birth order, delivery type, and gestational age), and quality of early childhood home environment.^37^ We removed one observation that was the only observation in a level of a categorical variable. This resulted in a final analysis sample of 350. Additional details on data are in Appendix S1.

#### Analysis methods

We fit four BKMR models: standard BKMR without modification (referred to as the exposure-only model), modifier-in-kernel, group-separable approach, and stratified. We used BKMR default priors presented in Appendix S1 for all models and tuned hyper-parameters to improve convergence of the MCMC algorithm. We assessed MCMC performance and convergence with effective sample sizes, Geweke statistics, and trace plots (effective sample size and Geweke statistics are available in Table S1 in Appendix S2).

We compare estimates of the exposure–response surface along with group-specific effects and between-group differences in effect estimates among the four models. We estimated the single-exposure effects for an interquartile range (IQR) change (${q}_1=\mathrm{0.25,}{q}_2=\mathrm{0.75}$) in each exposure while the others were fixed at each the median ($q=\mathrm{0.5}$). We estimated the total mixture effects by comparing the expected outcome at both the first and third quartiles (${q}_2=\mathrm{0.25,0.75}$) as compared to that at the median (${q}_1=\mathrm{0.5}$).

### Simulation methods

We evaluated the operating characteristics of the stratified and modifier-in-kernel methods and novel group-separable method. We compared performance of these models along with a standard BKMR model without modification (exposure-only). We consider scenarios with a two-level and a three-level modifying factor and scenarios where there is an effect only among one subgroup and when the exposure effects are similarly shaped by scaled differently among the groups.

To ensure a realistic correlation structure in the simulated data, we used the real exposures and modifier from the Bangladesh cohort in our simulation study. We included all $M=\mathrm{3}$ metal exposures. Of the three exposures, we simulated an association between only two of the exposures and the response. We considered three scenarios: nonlinear effect of two metals with interaction for boys ($n=\mathrm{179}$) and a similarly shaped but lower magnitude effect for girls ($n=\mathrm{171}$) (two-level scenario A), nonlinear effect of two metals with interaction for boys and no effect for girls (two-level scenario B), and nonlinear effect of two metals with interaction for group one ($n=\mathrm{45}$), a similarly shaped but lower magnitude effect for group two ($n=\mathrm{232}$), and no exposure effect for group three ($n=\mathrm{73}$) (three-level scenario). Full details are included in Appendix S1 and a visualization of the simulated exposure-response functions are in Figure S1.

We used the covariates from the Bangladesh cohort data set and generated their regression coefficients from a standard normal distribution. We simulated 1000 data sets for each scenario. The priors and MCMC specifications are listed in Appendix S1. We assessed MCMC performance and convergence with effective sample sizes, Geweke statistics, and trace plots (effective sample size and Geweke statistics are available in Tables S2 and S3 in Appendix S2).

We estimated the exposure–response surface across a grid of quantiles ranging from the first to third quartiles. We estimated total mixture effects across that same grid of quantiles for ${q}_2$ relative to median (${q}_1=\mathrm{0.5}$). We estimated single-exposure effects for an IQR change (${q}_1=\mathrm{0.25,}{q}_2=\mathrm{0.75}$) in each metal while the others were fixed ($q=\mathrm{0.25,0.5,0.75}$). We estimated the single-exposure and total mixture effects for each group and differences in the single-exposure and total mixture effects among groups.

To assess performance, we computed root mean squared error (RMSE) of the posterior mean along with coverage and average width for 95% credible intervals (CrIs) for each parameter. We averaged these metrics across exposure ranges defined in Appendix S1. We averaged RMSE across the modifier groups for group-specific effects and the exposure–response surface. We averaged metrics for each effect across the 1000 data sets.

## Results

### Analysis results

**Figure 1:**
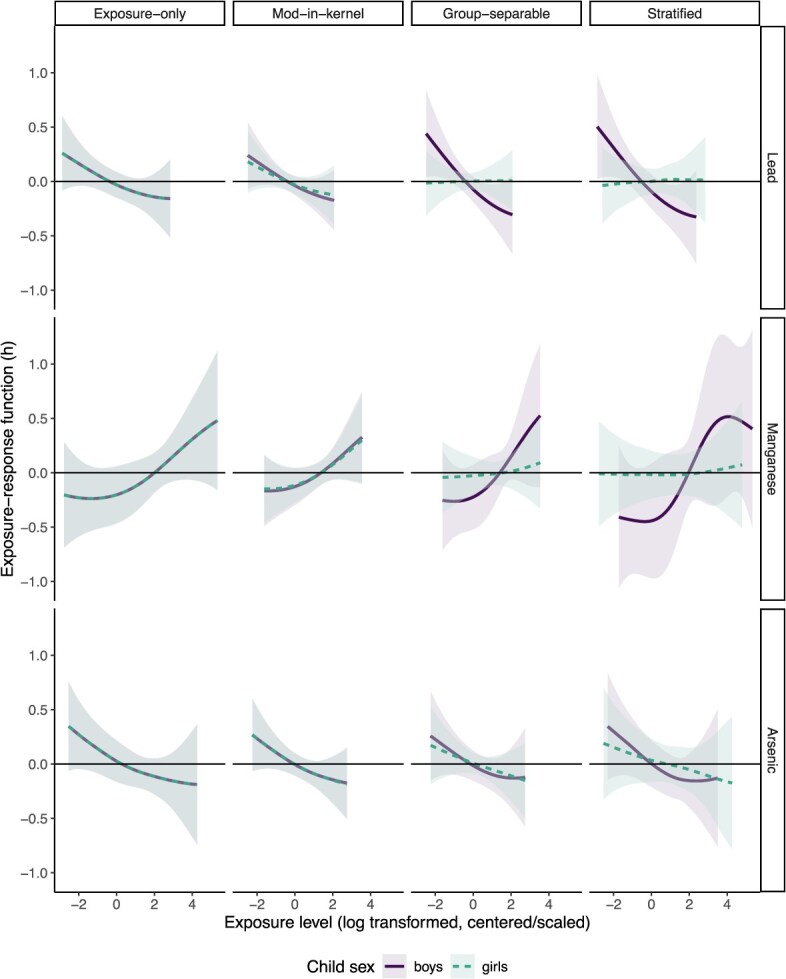
Estimated sex-specific single-exposure exposure–response functions for each metal exposure (rows) and each model (columns) in $n=\mathrm{350}$ children in the Bangladesh cohort. The line represents the posterior mean response for an individual in the specified group as exposure to one metal varies (x-axis) and the other metals are fixed at their medians. The ribbons represent 0.95 CrIs for the curve, and GS indicated the group-separable model.

**Figure 2:**
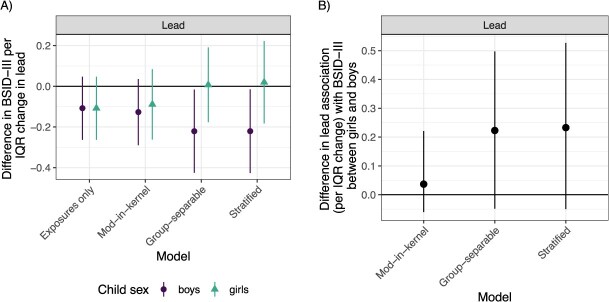
Estimated sex-specific and between-group differences in single-exposure effects of lead on BSID-III from each model (x-axis) in $n=\mathrm{350}$ children in the Bangladesh cohort. Panel a (left) shows the estimated average change in BSID-III for an IQR change in lead, while arsenic and manganese are fixed at their medians, for both girls (green triangles) and boys (purple circles) separately. Panel b (right) shows the estimated difference in this effect between girls and boys.

The analysis includes 179 boys and 171 girls. Figure 1 shows the estimated sex-specific exposure–response functions from each of the four models for each metal. Figure 2 shows estimated sex-specific single-exposure effects and estimated between-sex differences in single-exposure effects for lead.

All model estimates of the exposure–response function for lead in Figure 1 showed evidence that higher lead exposure is associated with lower cognitive performance. For the exposure-only model, this relationship was for both sexes combined because the exposure-only model only includes child sex as a covariate. Estimates of the exposure–response function from the modifier-in-kernel model were similar to estimates from the exposure-only model for both sexes, with almost no difference between the two estimated sex-specific exposure–response functions. In contrast, the estimates from the group-separable and stratified models demonstrated evidence of a negative relationship between lead and BSID-III for boys but no relationship for girls. These more flexible models had larger CrIs, with the stratified model having the largest CrIs.

For manganese, the estimated sex-specific exposure–response functions in Figure 1 from all models showed a positive relationship with BSID-III for boys. Overall, the CrIs are larger for manganese, providing little evidence of an exposure–response relationship statistically distinguishable from no effect. The estimated exposure–response functions from the modifier-in-kernel model were nearly identical for boys and girls. In contrast, the group-separable and stratified models showed evidence of effect modification. The estimated exposure–response functions for boys showed a positive relationship with BSID-III, while the estimated functions for girls did not show evidence of a relationship. For arsenic, the estimated functions from all models show no evidence of effect modification and flatter estimated exposure–response functions.

Figure 2 shows the sex-specific estimated single-exposure effect, or average difference in BSID-III per IQR change in lead (Figure 2A), and the estimated difference of that effect between boys and girls (Figure 2B) from each model. Figure 2A shows evidence that higher levels of lead are associated with lower cognitive function (lower BSID-III) from the group-separable and stratified models among boys, but no association among girls. Figure 2B shows moderate evidence of a difference in the lead association between boys and girls. Again, there are larger CrIs for the stratified model than the group-separable model. In contrast, we estimated smaller attenuated effects from the modifier-in-kernel and exposure-only models and no evidence of differences in these effects between boys and girls. For both manganese and arsenic, we found no evidence of sex-specific single-exposure effects on BSID-III (Figure S2), and we found no evidence of a difference in single-exposure effect between sexes (Figure S3 in Appendix A3). Furthermore, we found no evidence of an effect of the total mixture on BSID-III (Figures S4 and S5 in Appendix A3).

### Simulation results

Table 2 shows performance metrics for the four models we considered in the simulation study described above.

**Table 2:** Performance metrics for BKMR without the modifier (exposure-only), BKMR with the modifier in the kernel (mod-in-kernel), group-separable approach, and stratified models fit for 1000 simulated data sets across three simulation scenarios. Root mean squared error (RMSE), coverage (Cvg.), and average CrI width (width) were computed for each parameter estimate and averaged over the 1000 data sets.

**(lr)2-4 (lr)5-7 (lr)8-10 Model**	**2-Level Scenario A**			**2-Level Scenario B**			**3-Level Scenario**		
	**RMSE**	**Cvg.**	**Width**	**RMSE**	**Cvg.**	**Width**	**RMSE**	**Cvg.**	**Width**
Exposure–response surface									
Exposure-only	0.15	0.94	0.61	0.24	0.84	0.65	0.20	0.85	0.56
Mod-in-kernel	0.13	0.98	0.74	0.16	0.97	0.86	0.18	0.97	0.84
Group-separable	0.13	0.99	0.78	0.12	1.00	0.87	0.15	1.00	1.04
Stratified	0.13	0.99	0.82	0.13	1.00	0.91	0.18	0.99	1.19
Group-specific total mixture effect									
Exposure-only	0.19	0.70	0.32	0.32	0.44	0.34	0.25	0.51	0.29
Mod-in-kernel	0.15	0.81	0.37	0.19	0.81	0.44	0.22	0.69	0.37
Group-separable	0.13	0.87	0.39	0.13	0.93	0.42	0.15	0.93	0.49
Stratified	0.14	0.88	0.43	0.13	0.94	0.47	0.17	0.94	0.62
Group-specific single-exposure effect									
Exposure-only	0.28	0.82	0.72	0.43	0.66	0.73	0.37	0.67	0.63
Mod-in-kernel	0.25	0.88	0.81	0.31	0.88	0.95	0.34	0.79	0.80
Group-separable	0.25	0.91	0.88	0.24	0.95	0.94	0.29	0.94	1.11
Stratified	0.26	0.90	0.96	0.25	0.95	1.03	0.34	0.95	1.43
Between-group differences in total mixture effect									
Mod-in-kernel	0.19	0.64	0.34	0.30	0.65	0.52	0.32	0.37	0.35
Group-separable	0.12	0.96	0.54	0.16	0.92	0.59	0.19	0.95	0.70
Stratified	0.13	0.96	0.61	0.17	0.93	0.67	0.21	0.96	0.93
Between-group differences in single-exposure effect									
Mod-in-kernel	0.27	0.77	0.71	0.46	0.76	1.04	0.44	0.58	0.69
Group-separable	0.25	0.96	1.18	0.33	0.93	1.30	0.35	0.96	1.49
Stratified	0.29	0.97	1.35	0.35	0.94	1.47	0.42	0.97	2.03

#### ***Exposure***–***response surface***

For the estimated exposure–response surface across all three scenarios, the group-separable BKMR model had RMSE as low or lower than the other models. RMSE for the stratified model was similar to RMSE for the group-separable model in the two-level scenarios but larger in the three-level scenario. Across all scenarios, coverage was similar for the three models that included the modifier but lower for the exposure-only model. However, the average CrI widths varied substantially. The exposure-only and modifier-in-kernel models had small average CrI widths, and the stratified model had the largest average CrI widths.

#### Group-specific effects

For both the group-specific total mixture effect estimates and single-exposure effect estimates, the group-separable model had RMSE as low or lower than the other models. The group-separable and stratified models had similar RMSE in the two-level scenarios, but the group-separable model had lower RMSE in the three-level scenario. The group-separable and stratified models had similar coverage, but the group-separable model had smaller average CrI widths. For group-specific single-exposure effect estimates in the three-level scenario, average CrI width for the group-separable model was 1.11, but average CrI width for the stratified model was 1.43.

The modifier-in-kernel and exposure-only models generally had higher RMSE than the group-separable model and had coverage well below the nominal coverage level. For example, in two-level scenario B, the total mixture effect RMSE was higher for the exposure-only (0.32) and modifier-in-kernel (0.19) models than for the group-separable and stratified models (both 0.13). Coverage for the exposure-only and modifier-in-kernel models for the single exposure and total mixture effects ranged from 0.44 to 0.88, while the group-separable and stratified were higher in each setting and mostly near the nominal level of 0.95.

#### Between-group differences in effects

For the between-group differences in both the total mixture effect and the single-exposure effect estimates, the group-separable model had RMSE as low or lower than the stratified model. While the group-separable and stratified models had similar coverage, the group-separable model had smaller CrI widths on average compared to the stratified model. The modifier-in-kernel model had higher RMSE than the group-separable model and had coverage below the nominal level (0.37-0.77). The exposure-only model cannot estimate between-group differences.

## Discussion

There is substantial interest in estimating the health effects of environmental mixtures, and BKMR has emerged as a popular tool for such analyses. However, there is little guidance on how to conduct subgroup analyses and estimate between-group differences in effects with BKMR. In this paper, we presented and evaluated methods for assessing effect modification by a categorical variable with BKMR. The first method we considered fits multiple BKMR models stratified by the levels of the modifier. The second method includes the modifier directly into the kernel function used to model the exposure–response function. These first two methods can be implemented with the existing BKMR framework and software. The other method we consider is a new variant of the BKMR framework that involves constructing a group-separable kernel. This structure allows for increased flexibility in the group-specific exposure–response functions while allowing for coherent inference on both subgroup-specific effects and between-group differences.

Table 1 highlights the differences in assumptions for each approach. The least flexible model is the modifier-in-kernel model, which includes the modifier in the kernel of a standard BKMR model along with the exposures. This approach shares all model parameters among groups, including smoothing parameters for the exposure–response functions, the covariates' effects, and residual variance. Including the modifier in the kernel this way forces a similar shape in the exposure–response function for all groups. At the other extreme is the stratified BKMR approach, which fits multiple models stratified by the modifier levels. Stratified BKMR does not share any parameters across groups. While this provides the most flexibility in allowing for effect modification, not sharing information across groups can increase the variance of the estimators and limit inferences that can be made. This is especially true when some levels of the modifier have small sample sizes or the effects are quite similar across subgroups. The new model fits BKMR with a separable covariance function. This pools observations and shares exposure-specific smoothing, covariate effects, and residual variance parameters across groups. The tradeoff between assumptions of existing models allows the new model to address limitations of existing approaches.

The results from the analysis of the Bangladesh cohort illustrate the differences in these methods. Using the modifier-in-kernel method, we estimated exposure–response functions for each metal that were nearly identical for both boys and girls. In contrast, we estimated different exposure–response functions for girls and boys using the group-separable and stratified models. The results demonstrate the progression of these methods where the more flexible methods have increased capacity to estimate group-specific effects and between-group differences but have larger variances.

Results from the simulation study demonstrate similar differences among the methods. In most scenarios, the modifier-in-kernel model had RMSE similar to, or larger than, the group-separable model, and often had coverage below the nominal level and narrower intervals. This indicates that while the model had lower variance, RMSE was larger due to increased bias that resulted from the restrictive nature of the imposed penalty, which forces the exposure–response surface to be similar between groups. In fact, the modifier-in-kernel model often performed similarly to the exposure-only model, which did not include modification. Similarly, the stratified model had larger RMSE than the group-separable model, but while coverage was close to the nominal level, CrIs were wider. This combination of lower RMSE but increased CrI widths demonstrates a bias-variance tradeoff between the methods where the group-separable BKMR model has lower bias but larger variance than the modifier-in-kernel method. The group-separable model had comparatively low RMSE and smaller interval widths compared to both the modifier-in-kernel and stratified approaches. The reduction in variance compared to the stratified model is most apparent for estimates of between-group differences in effects and in the three-level scenario due to smaller sample size in each stratified model. This implies that, relative to the other methods, both the bias and variance are low using the group-separable approach.

The group-separable model introduced here is the “Goldilocks” model, combining the lower variance of a pooled analysis that uses parameter sharing with the flexibility in the exposure–response functions of a stratified model. Because the group-separable model does not impose penalization on the shape of the exposure–response surface among the groups, the model has increased flexibility to better estimate group-specific effects and between-group differences. Due to parameter sharing among the groups, the group-separable model had lower variance than the stratified model, particularly when some subgroups had small sample sizes.

There are limitations and areas left unexplored by this work, many of which could be the target for future research. There may be additional alternative parameterizations of BKMR that perform differently for estimating subgroup-specific effects and between group differences. We explored a less flexible version that has a shared smoothing parameter $\tau$ and a heterogeneous model with group-specific error variance (${\sigma}_{w_i}$). Both approaches are available in the bkmrGS package. We found no evidence that a shared $\tau$ improved performance in simulation or data analysis. Allowing for heteroskedasticity had no impact on the data analysis results, and we did not test any simulation settings with heteroskedasticity. A second limitation is that we did not explore the operating characteristics of exposure selection in this framework. Selection of exposures could be incorporated, but it is unclear whether selection should be applied overall or separately for each group. Other hierarchical structures on group-specific variables could be explored as well. Our work only considers a single categorical modifier, but heterogeneity may be attributed to multiple variables. Extending the methods presented here to multiple modifiers would require alternative methods similar to those developed in other settings.^38^ Finally, we relied on the original MCMC approach for BKMR,^32^^,^^33^ which requires inverting a $n\times n$ matrix, where $n$ is the sample size. The group-separable model only requires inverting matrices the size of the subgroups. This could be exploited for substantial gains in computing. In addition, there is active research into improved MCMC for BKMR that could be incorporated into these methods.

In light of our simulation and data analysis results, we recommend using our group-separable model to include categorical effect modification in BKMR. For studies with a large sample sizes within each subgroup, the stratified model is also a good choice and can be expected to perform similarly to the group-separable model. For smaller sample sizes or samples with some small subgroups, the parameter sharing of the group-separable model will result in better performance. We do not recommend the modifier-in-kernel approach.

## Supplementary Material

Web_Material_kwaf281

## Data Availability

The R package for the group-separable BKMR method is available at https://github.com/ddemateis/bkmrGS. All data and code to reproduce the results are available at https://github.com/ddemateis/bkmr_simulations.
