# Supplementary material for: Subgroup analyses and effect modification with Bayesian kernel machine regression
Source: Am J Epidemiol. 2025 Dec 19;195(4):1154–62. doi: 10.1093/aje/kwaf281 (PMC12758637; doi:10.1093/aje/kwaf281)
Supplement: Web_Material_kwaf281 [file web_material_kwaf281.pdf]

Supplement to "Subgroup Analyses and Effect Modification with Bayesian Kernel Machine Regression"

Danielle Demateis[1], Kayleigh P. Keller[1], Brent A. Coull[2], and Ander Wilson[1]

[1]Department of Statistics, Colorado State University, Fort Collins, CO, USA

[2]Department of Biostatistics, Harvard T. H. Chan School of Public Health, Boston, MA, USA

CONTENTS

APPENDIX S1: ADDITIONAL METHODS

Interpretation of the Exposure-Response Function

Simulation Design

MCMC and prior specification for models

Simulation Methods

APPENDIX S2: ADDITIONAL TABLES

Table S1: Effective sample size (ESS) and Geweke statistics for BKMR without the modifier (exposure-only), BKMR with the modifier in the kernel (mod-in-kernel), group-separable approach, and stratified models fit for the data analysis presented in the main text. ESS and GWK were computed for the exposure-response surface while with all exposures fixed at the following quantiles: 0, 0.05, 0.25, 0.5, 0.75, 0.95, 1.

Table S2: Effective sample size (ESS) for BKMR without the modifier (exposure-only), BKMR with the modifier in the kernel (mod-in-kernel), group-separable approach, and stratified models fit for 1000 simulated data sets across three simulation scenarios presented in the main text. ESS was computed for the exposure-response surface while with all exposures fixed at their minimums, maximums, and 5th, 25th, 50th, 75th, and 95th percentiles.

Table S3: Geweke statistic for BKMR without the modifier (exposure-only), BKMR with the modifier in the kernel (mod-in-kernel), group-separable approach, and stratified

models fit for 1000 simulated data sets across three simulation scenarios presented in the main text. GWK was computed for the exposure-response surface while with all exposures fixed at their minimums, maximums, and 5th, 25th, 50th, 75th, and 95th percentiles.

APPENDIX S3: ADDITIONAL FIGURES

Figure S1: Exposure-response surfaces used in simulation. The axes on the horizontal plane are the first two exposures, and the vertical axis is the mean response. The exposure-response surface is constant across the third exposure.

Figure S2: Estimated sex-specific single-exposure effects of lead, manganese, and arsenic on BSID-III from each model (x-axis) in $n = 350$ children in the Bangladesh cohort. This is the estimated average change in BSID-III for an IQR change in exposure, while other exposures are fixed at their medians, for both girls (green triangles) and boys (purple circles) separately.

Figure S3: Estimated between-group differences (girls effect minus boys effect) in single-exposure effects of lead, manganese, and arsenic on BSID-III from each model (x-axis) in $n = 350$ children in the Bangladesh cohort. This is the estimated difference between girls and boys average change in BSID-III for an IQR change in exposure, while other exposures are fixed at their medians.

Figure S4: Estimated sex-specific total effects of the mixture on BSID-III from each model (x-axis) in $n = 350$ children in the Bangladesh cohort. This is the estimated average change in neurodevelopment score for a change in all exposures between their first quartile and median (facet 1) and between their third quartile and median (facet 2) for girls (green triangles) and boys (purple circles).

Figure S5: Estimated differences (girls effect minus boys effect) between girls and boys in total effects of the mixture on BSID-III from each model (x-axis) in $n = 350$ children in the Bangladesh cohort. This is the estimated difference girls and boys average change in neurodevelopment score for a change in all exposures between their first quartile and median (facet 1) and between their third quartile and median (facet 2).

APPENDIX S1: ADDITIONAL METHODS

Interpretation of the Exposure-Response Function

For a standard BKMR model without modification, the total effect of the mixture on the response is the difference in mean outcome when all exposures are fixed at a specific quantile (q_1) compared to when all exposures are fixed at a different quantile (q_2). Let z_m^q denote the qth quantile of $[z_{1m}, \ldots, z_{nm}]'$. Formally, the total mixture effect is $\Delta_{tot}(q_1, q_2) = h(z_1^{q_2}, \ldots, z_M^{q_2}) - h(z_1^{q_1}, \ldots, z_M^{q_1})$. For a model with modification, the total mixture effect specific to the pth modifier group ($w = p$) is $\Delta_{tot}(q_1, q_2|p) = h(z_1^{q_2}, \ldots, z_M^{q_2}|w = p) - h(z_1^{q_1}, \ldots, z_M^{q_1}|w = p)$. Additionally, the between-group difference of the total mixture effect between modifier groups p and p' is the change in total mixture effect between modifier groups p and p', i.e., $\Delta_{tot}(q_1, q_2|p') - \Delta_{tot}(q_1, q_2|p)$.

For a standard BKMR model without modification, the single-exposure effect of a given exposure on the response is the difference in mean outcome between when one exposure is fixed at the q_1 quantile versus when it is fixed at the q_2 quantile and all other all other exposures are fixed at q. For example, the single-exposure effect of $\mathbf{z}_1$ is $\Delta_1(q_1, q_2, q) = h(z_1^{q_2}, z_2^q, \ldots, z_M^q) - h(z_1^{q_1}, z_2^q, \ldots, z_M^q)$. For a model with modification, the single-exposure effect of a given exposure on the response in a modifier group $w = p$ is the change in the mean outcome between quantiles q_1 and q_2 for that exposure, while all other exposures are fixed at q and only the exposure-response surface for modifier group $w = p$ is considered. For example, the single-exposure effect of the first exposure in modifier group p is $\Delta_1(q_1, q_2, q|p) = h(z_1^{q_2}, z_2^q, \ldots, z_M^q|w = p) - h(z_1^{q_1}, z_2^q, \ldots, z_M^q|w = p)$. The between-group difference for the single-exposure effect between modifier levels p and p' is $\Delta_1(q_1, q_2, q|p') - \Delta_1(q_1, q_2, q|p)$.

To estimate between-group differences in effects using the stratified model, we matched posterior samples from each modifier group and performed inference directly on those matched posterior samples.

Simulation Design

Simulation surfaces were generated using the following equations: $h_1(\mathbf{z}) = 4f[\frac{1}{4}(\Sigma_{m=1}^{2} z_m + \frac{1}{2}\prod_{m=1}^{2} z_m), 0, 0.3]$, $h_2 = \frac{1}{4}h_1$, and $h_3(\mathbf{z}) = 0$. The function f is the logistic distribution $f(x, \mu, \sigma) = \frac{1}{\sigma}\exp[(x-\mu)/\sigma]/[1 + \exp\{(x-\mu)/\sigma\}]^2$.

Figure S1 shows the three exposure-response surfaces that we used to construct modification scenarios. Simulation surfaces were generated using the following equations: $h_1(\mathbf{z}) = 4f[\frac{1}{4}(\Sigma_{m=1}^{2} z_m + \frac{1}{2}\prod_{m=1}^{2} z_m), 0, 0.3]$, $h_2 = \frac{1}{4}h_1$, and $h_3(\mathbf{z}) = 0$. The function f is the logistic distribution $f(x, \mu, \sigma) = \frac{1}{\sigma}\exp[(x-\mu)/\sigma]/[1 + \exp\{(x-\mu)/\sigma\}]^2$. We assume that the three surfaces h_1, h_2, and h_3 are constant across the third exposure.

To ensure some correlation between the modifier and other variables, we used child sex as the modifier in the 2-level scenarios and one of the HOME score components as the modifier in the 3-level scenario (and thus did not include HOME score components as covariates in this model). The scenarios are as follows.

1. **2-Level Scenario A**: The outcomes in the boy group ($n = 179$) are simulated from exposure-response surface h_1, and the outcomes in the girl group ($n = 171$) are simulated from exposure-response surface h_2, which has less of an effect.

2. **2-Level Scenario A**: The outcomes in the boy group are simulated from exposure-response surface h_1. The observations the girl group are simulated from exposure-response surface h_3, which has no effect.

3. **3-Level Scenario**: The outcomes in group one ($n = 45$) are simulated from exposure-response surface h_1, the observations in group two ($n = 233$) are simulated from exposure-response surface h_2, and the observations in group three are simulated from h_3 ($n = 73$).

The main effect for each modifier group, with respect to the reference group, was 1. To generate response values, we used a Gaussian likelihood with variance σ^2 and used a signal-

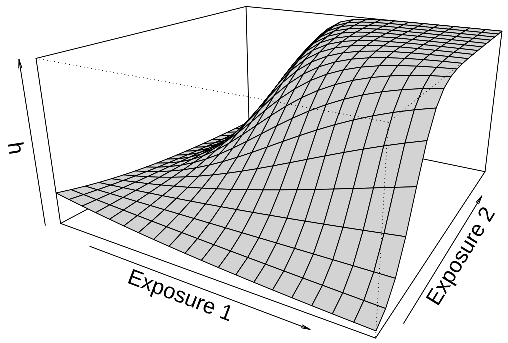

(a) h_1

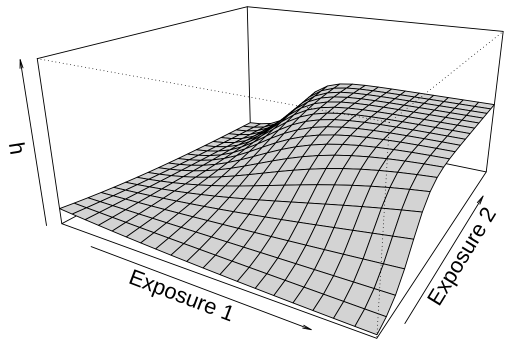

(b) h_2

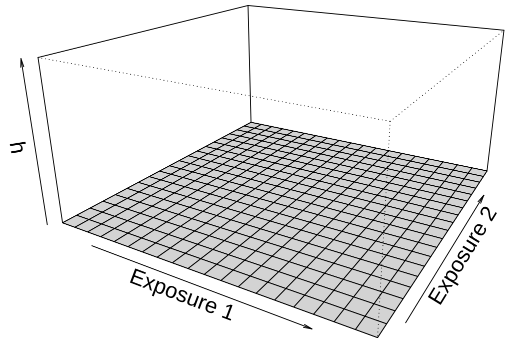

(c) h_3

Figure S1: Exposure-response surfaces used in simulation. The axes on the horizontal plane are the first two exposures, and the vertical axis is the mean response. The exposure-response surface is constant across the third exposure.

to-noise ratio of 1, where signal is the standard deviation of the mean response $\mathbf{h} + \mathbf{X}\boldsymbol{\beta}$ and noise is σ.

MCMC and prior specification for models

We used the same prior distributions and hyperparameters for all models considered. We used a flat prior on the regression coefficients $\boldsymbol{\beta}$ and an inverse Gamma prior on the error variance, $\sigma^2 \sim \text{IG}(0.001, 0.001)$. Following Bobb et al.[1], we parameterized $\lambda = \tau\sigma^{-2}$ and assigned a Gamma prior with mean 100 and variance 100. We used the same priors for the group-specific λ parameters in the second group-separable model. Finally, the smoothing parameters ρ_m for each exposure $m = 1, \ldots, M$ has prior $\rho_m \sim \text{Uniform}(0, 100)$. We used the same priors for ρ_p in the modifier-in-kernel model.

For the data analysis, we obtained 25,000 posterior samples by running the exposure-only, group-separable, and stratified models for 270,000 iterations and discarded 20,000 iterations for warm-up and the modifier-in-kernel model for 320,000 iterations and discarded 70,000 for warm-up, and thinned by 10. We chose warm-up periods based on visual assessments of trace plots for parameters, and we thinned to reduce postprocessing computation time.

For the simulation, we tuned hyper-parameters, i.e., step size and starting values, for model convergence. We ran all models for 15,000 MCMC iterations, removing 5,000 as warm-up and thinned by 5.

Simulation Methods

We consider different exposure ranges for each effect estimate to prevent extrapolation. For group-specific effect estimates, we evaluate performance in each group only over the range of exposures in that group. For between-group differences, we evaluate performance using the intersection of the range of exposures observed in both groups of the difference.

APPENDIX S2: ADDITIONAL TABLES

Table S1: Effective sample size (ESS) and Geweke statistics for BKMR without the modifier (exposure-only), BKMR with the modifier in the kernel (mod-in-kernel), group-separable approach, and stratified models fit for the data analysis presented in the main text. ESS and GWK were computed for the exposure-response surface while with all exposures fixed at the following quantiles: 0, 0.05, 0.25, 0.5, 0.75, 0.95, 1.

	Exposures only		Mod-in-kernel		Group-separable		Stratified	
Quantile	ESS	Geweke	ESS	Geweke	ESS	Geweke	ESS	Geweke
				Girls				
0	4785	-1.52	4766	-0.19	6564	-0.14	23437	-0.02
0.05	3746	-1.36	3746	0.14	6945	0.36	23180	0.28
0.25	2364	-1.36	3069	0.16	6620	0.61	21673	0.66
0.5	2081	-1.43	2842	0.11	6108	0.53	20270	0.76
0.75	1887	-1.58	2729	0.05	5603	0.33	19584	0.56
0.95	1793	-1.74	2717	0.01	4829	-0.10	16953	-0.33
1.00	3989	-0.86	4701	-0.06	8650	0.40	19509	-0.68
				Boys				
0	5066	-1.33	5867	-0.03	6711	0.63	23074	0.17
0.05	3424	-1.25	4654	0.18	6156	0.64	23706	0.48
0.25	2347	-1.29	3291	0.19	6044	0.51	21307	0.55
0.5	2149	-1.34	2903	0.15	6019	0.45	19910	0.62
0.75	1882	-1.51	2667	0.11	5876	0.44	18709	0.51
0.95	1789	-1.68	2690	0.06	5829	0.64	17679	0.49
1.00	6242	-0.64	6757	0.15	10270	-0.04	24107	0.25

Table S2: Effective sample size (ESS) for BKMR without the modifier (exposure-only), BKMR with the modifier in the kernel (mod-in-kernel), group-separable approach, and stratified models fit for 1000 simulated data sets across three simulation scenarios presented in the main text. ESS was computed for the exposure-response surface while with all exposures fixed at their minimums, maximums, and 5th, 25th, 50th, 75th, and 95th percentiles.

Model	Min	5th	25th	50th	75th	95th	Max
2-Level Scenario A							
Exposure-only	1557	1547	1515	1518	1506	1457	1361
Mod-in-kernel	1502	1470	1447	1450	1442	1380	1255
Group-separable	1558	1532	1507	1505	1501	1457	1313
Stratified	1594	1606	1621	1617	1606	1562	1443
2-Level Scenario B							
Exposure-only	1410	1429	1436	1450	1429	1442	1480
Mod-in-kernel	1360	1369	1379	1384	1363	1301	1270
Group-separable	1525	1535	1524	1515	1505	1476	1371
Stratified	1538	1533	1520	1516	1510	1500	1500
3-Level Scenario							
Exposure-only	1118	1091	1055	1050	1025	906	791
Mod-in-kernel	1027	983	959	946	927	814	750
Group-separable	1365	1341	1292	1263	1242	1183	1162
Stratified	1428	1400	1348	1344	1319	1248	1278

Table S3: Geweke statistic for BKMR without the modifier (exposure-only), BKMR with the modifier in the kernel (mod-in-kernel), group-separable approach, and stratified models fit for 1000 simulated data sets across three simulation scenarios presented in the main text. GWK was computed for the exposure-response surface while with all exposures fixed at their minimums, maximums, and 5th, 25th, 50th, 75th, and 95th percentiles.

Model	Min	5th	25th	50th	75th	95th	Max
2-Level Scenario A							
Exposure-only	0.21	0.14	0.05	0.01	-0.03	-0.14	-0.27
Mod-in-kernel	0.10	0.06	0.01	-0.01	-0.03	-0.09	-0.14
Group-separable	-0.08	-0.10	-0.11	-0.11	-0.10	-0.10	-0.10
Stratified	0.08	0.07	0.04	0.01	-0.02	-0.08	-0.13
2-Level Scenario							
Exposure-only	0.25	0.22	0.11	0.05	-0.00	-0.12	-0.13
Mod-in-kernel	0.12	0.02	-0.10	-0.13	-0.19	-0.25	-0.30
Group-separable	0.01	0.01	0.00	0.01	0.01	0.01	-0.01
Stratified	0.04	0.04	0.03	0.01	-0.01	-0.06	-0.09
3-Level Scenario							
Exposure-only	-0.22	-0.28	-0.40	-0.48	-0.57	-0.75	-0.81
Mod-in-kernel	-0.06	-0.06	-0.09	-0.12	-0.16	-0.24	-0.28
Group-separable	-0.00	-0.01	-0.01	-0.01	-0.01	-0.00	-0.01
Stratified	0.08	0.06	0.02	-0.03	-0.07	-0.11	-0.14

APPENDIX S3: ADDITIONAL FIGURES

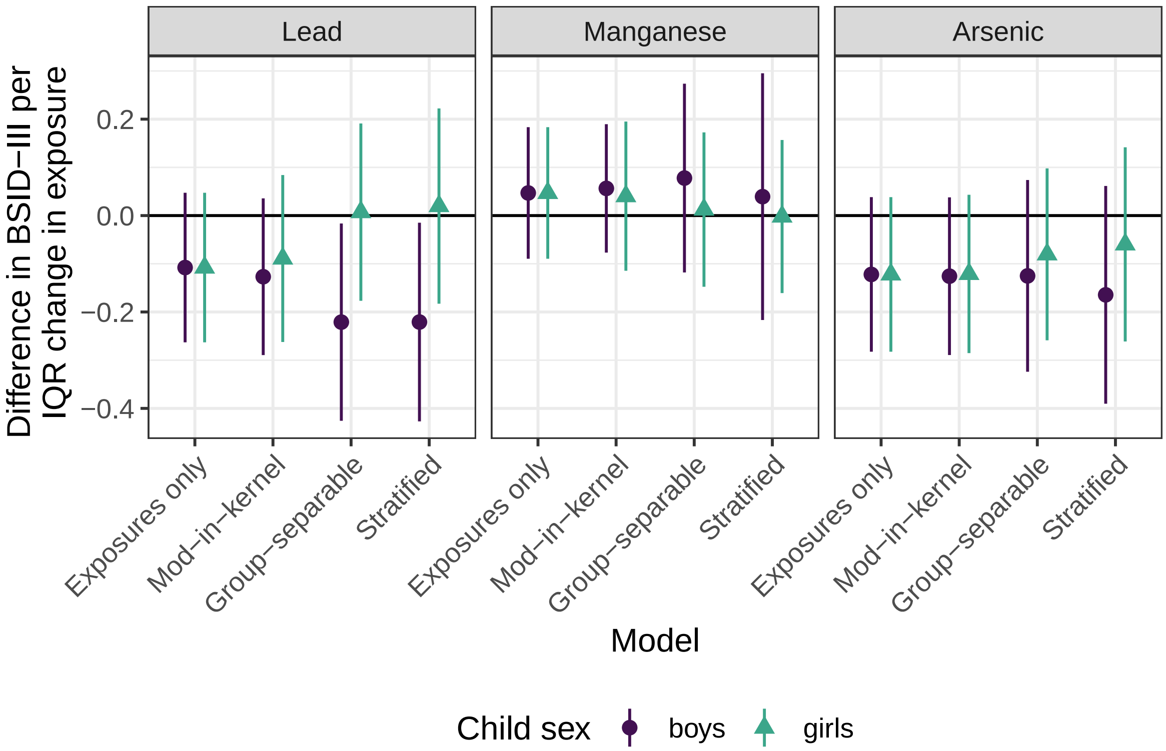

Figure S2: Estimated sex-specific single-exposure effects of lead, manganese, and arsenic on BSID-III from each model (x-axis) in $n = 350$ children in the Bangladesh cohort. This is the estimated average change in BSID-III for an IQR change in exposure, while other exposures are fixed at their medians, for both girls (green triangles) and boys (purple circles) separately.

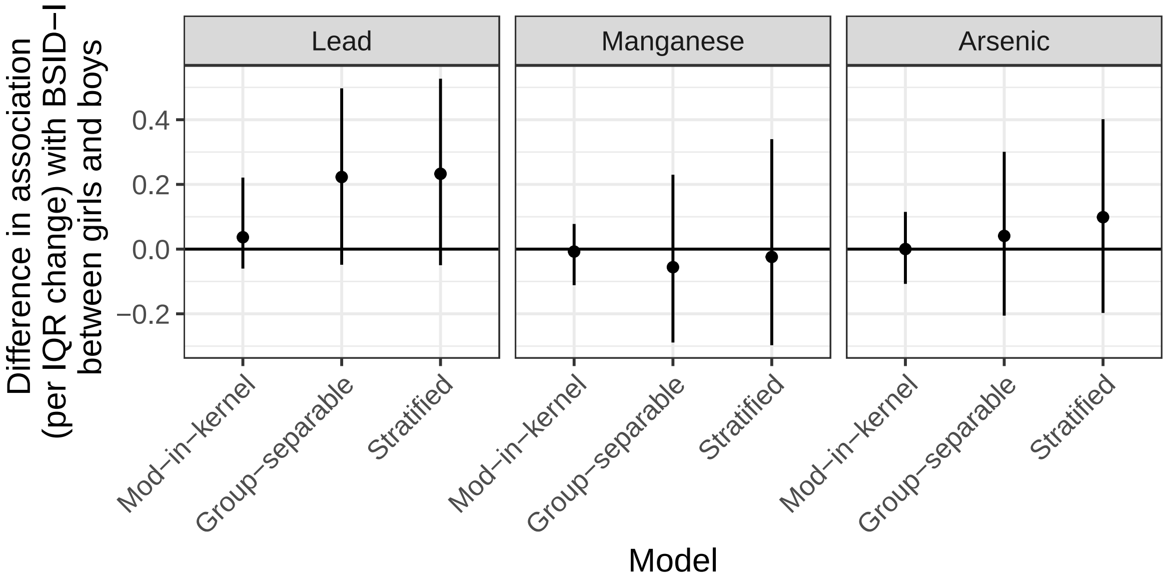

Figure S3: Estimated between-group differences (girls effect minus boys effect) in single-exposure effects of lead, manganese, and arsenic on BSID-III from each model (x-axis) in $n = 350$ children in the Bangladesh cohort. This is the estimated difference between girls and boys average change in BSID-III for an IQR change in exposure, while other exposures are fixed at their medians.

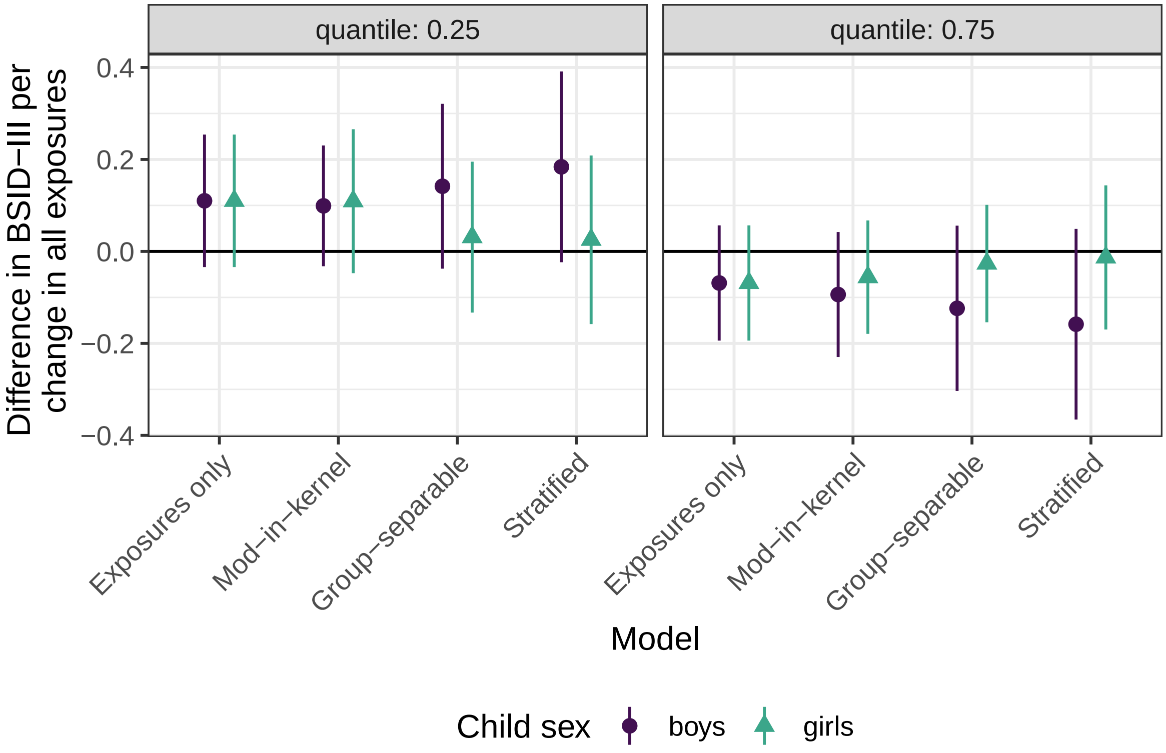

Figure S4: Estimated sex-specific total effects of the mixture on BSID-III from each model (x-axis) in $n = 350$ children in the Bangladesh cohort. This is the estimated average change in neurodevelopment score for a change in all exposures between their first quartile and median (facet 1) and between their third quartile and median (facet 2) for girls (green triangles) and boys (purple circles).

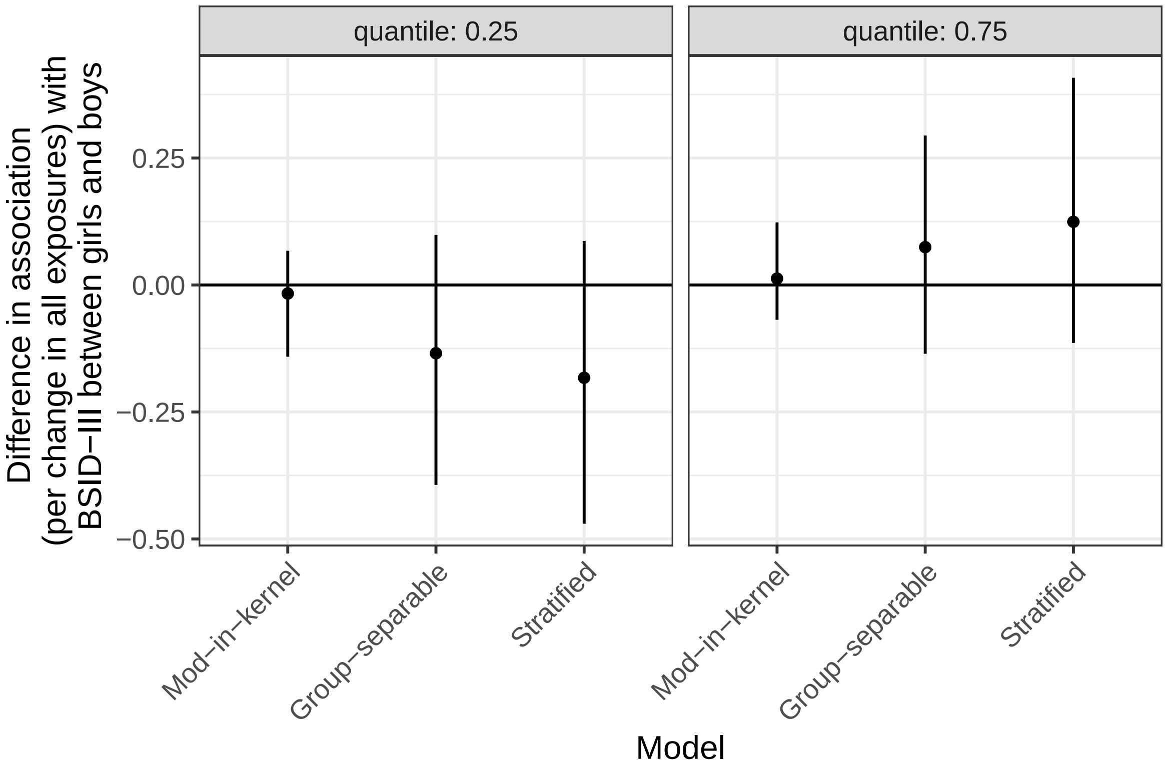

Figure S5: Estimated differences (girls effect minus boys effect) between girls and boys in total effects of the mixture on BSID-III from each model (x-axis) in $n = 350$ children in the Bangladesh cohort. This is the estimated difference girls and boys average change in neurodevelopment score for a change in all exposures between their first quartile and median (facet 1) and between their third quartile and median (facet 2).

References

[1] Jennifer F. Bobb, Linda Valeri, Birgit Claus Henn, David C. Christiani, Robert O. Wright, Maitreyi Mazumdar, John J. Godleski, and Brent A. Coull. Bayesian kernel machine regression for estimating the health effects of multi-pollutant mixtures. *Biostatistics*, 16(3):493–508, July 2015. ISSN 1468-4357, 1465-4644. doi: 10.1093/biostatistics/kxu058. URL `https://academic.oup.com/biostatistics/article/16/3/493/269719`.
